# Points of view matter when assessing biodiversity vulnerability to environmental changes

**DOI:** 10.1111/gcb.15054

**Published:** 2020-03-23

**Authors:** Alejandro Ordonez

**Affiliations:** ^1^ Section for Ecoinformatics & Biodiversity Department of Biology Aarhus University Aarhus Denmark; ^2^ Center for Biodiversity Dynamics in a Changing World (BIOCHANGE) Aarhus University Aarhus Denmark; ^3^ University of Utrecht Copernicus Institute of Sustainable Development Utrecht The Netherlands

## Abstract

We can expect different levels of vulnerability depending on the paradigm used to determine the mechanisms that will alter biodiversity under climate change. A multi‐paradigm perspective is necessary to get the full picture of biodiversity vulnerability.

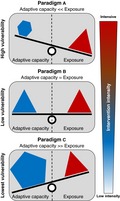

This is a commentary on https://doi.org/10.1111/gcb.15008.

The last three decades have seen an exponential growth in the number of studies evaluating where, when, why and how biodiversity could be affected by climate change and land degradation. Evidence for these effects comes from three distinct lines of research: (a) empirical and model‐based assessments of range shifts in species distributions linked to environmental changes (Chen, Hill, Ohlemuller, Roy, & Thomas, 2011); (b) compositional and richness changes (Blowes et al., 2019); and (c) links between life‐history, eco‐physiological or biogeographical attributes and the populations’ responses to environment changes (Angert et al., 2011). As the literature on environmental change impacts continues to grow, it is essential to translate the observed and expected patterns of biodiversity change to a metric that describes the threat to biodiversity from the adverse effects of environmental transformations. However, assessing biodiversity responses to observed or expected changes in environmental conditions is a multifaceted problem, where the point of view used to determine both the drivers and the degree of change matter. In this issue of *Global Change Biology*, Kling, Auer, Comer, Ackerly, and Hamilton (2020) tackle these problems by focusing on two of the facets (observed multivariate climate change and limiting environmental factors) defining how biodiversity may respond to climate change. Then they combine these facets to show how biodiversity could respond to novel climatic conditions under alternative paradigms of response.

Quantifying where environmental changes can be the most harmful to biodiversity is fundamental, not just for the development and implementation of successful mitigation and adaptation strategies to these changes, but also to understand the future of ecological dynamics in the Anthropocene. Given the evidence of wide variability in the magnitude and type of responses to environmental changes from populations, species, communities and ecosystems (Scheffers et al., 2016), assessing the threat to biodiversity requires appropriate framing. This framing means that any metric that describes biodiversity change requires a conceptual context that links environmental shifts to a possible response mechanism of the biological entity of interest. The idea of vulnerability (i.e. the susceptibility or ability to cope with the experienced or expected adverse effects of climate change; cf. McCarthy, Canziani, Leary, Dokken, & White, 2001) provides such a framing. It does so by linking the two main factors determining the susceptibility and ability to respond to changing environmental conditions (Figure 1). The first of these main factors are the external contributing factors that describe the magnitude of the drivers of change (exposure; red triangles in Figure 1). The second main factor includes the intrinsic contributing factors that describe the balance between the degree to which the survival or persistence of a species is contingent on the prevailing environmental setup (sensitivity) and the capacity of that species to resist environmental change (adaptive capacity; blue shapes in Figure 1).

**FIGURE 1:**
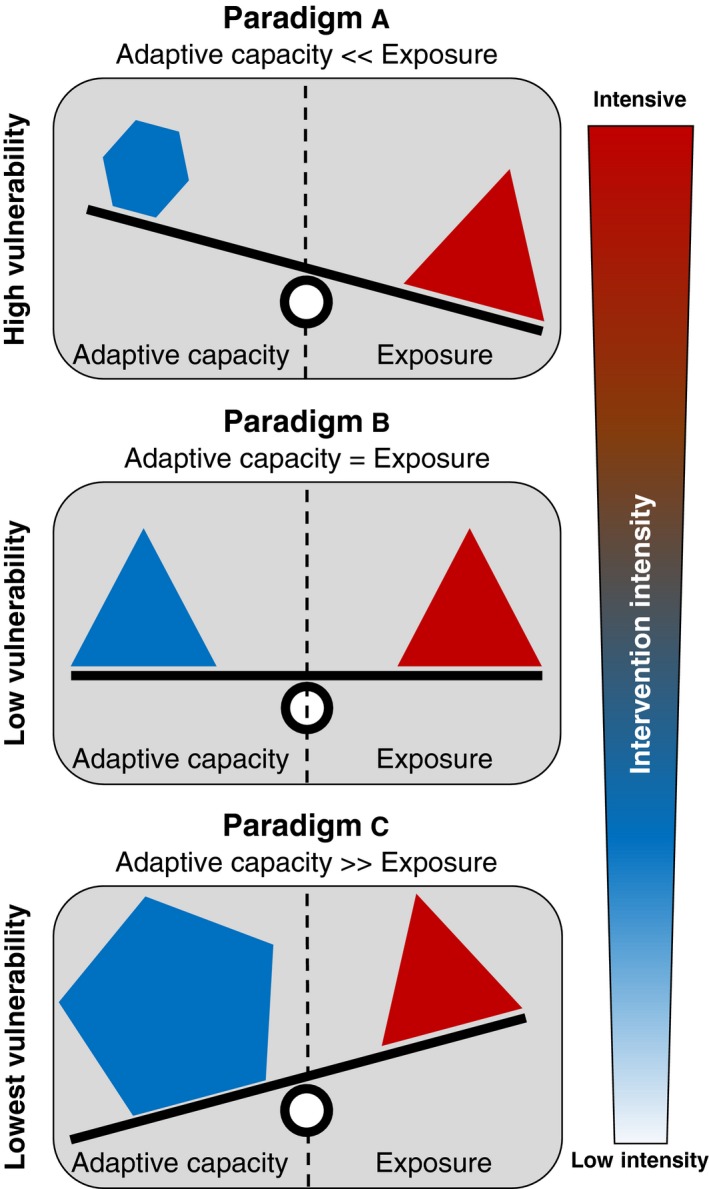
Importance of using the appropriate paradigm when defining the vulnerability of population, species, communities or ecosystems to environmental changes. The impact mechanism determining the paradigm of evaluation defines the adaptive capacity (i.e. the ability to accommodate, to adjust or resist to an impact; blue shapes) of the entity of interest. Under similar environmental exposure (magnitude of the drivers of change; red triangles) the paradigm used to define a population, species, communities or ecosystems would define the perceived vulnerability and therefore the required intervention intensity. Consideration of multiple mechanisms that will alter biodiversity under current and future environmental changes has an essential place in holistic vulnerability assessments and is critical for defining potential conservation, mitigation or adaptation actions

Assessing the vulnerability of populations, species, communities or ecosystems, although conceptually simple, is riddled with practical and conceptual complexities regarding how to measure the different aspects embedded in the vulnerability concept (Dawson, Jackson, House, Prentice, & Mace, 2011; Foden et al., 2019). Managing the complexities of measuring and linking measures of exposure, sensitivity and adaptive capacity could be achieved by framing their assessment within a well‐defined vulnerability paradigm which describes the response mechanism of the biological entity of interest (Figure 1). This perspective is the one taken by Kling et al. (2020). They propose three alternative vulnerability paradigms, each centred on an alternative definition of how biodiversity could respond to changing environmental conditions. Specifically, their work disaggregates the vulnerability question into three distinct conceptual paradigms of ecological response that reflect a balance between exposure and adaptive capacity. At the centre of each of the proposed paradigms of ecological response are alternative impact mechanisms by which environmental change could affect biodiversity. The first paradigm focuses on the role of the fundamental niche in determining the probability of a species persisting, or not, as climate changes (i.e. the niche novelty paradigm). The second paradigm focuses on assessing possible reductions in demographic performance as climate deviates from conditions that allow a species to persist at a location (i.e. the temporal novelty paradigm). The third, and last, paradigm focuses on assessing how fast a population would need to move to reach the most similar conditions as those experienced today (i.e. the spatial novelty paradigm). This focus on impact mechanisms is central for translating vulnerability assessments into actionable conservation, mitigations and adaptation plans. Furthermore, the results of Kling et al. (2020) show how a multi‐paradigm perspective is necessary to get the full picture of biological vulnerability to environmental change.

Translating vulnerability assessments into biodiversity conservation plans has been difficult due to the diversity of mechanisms that underlie the reported impact of the observed environmental forcing on biodiversity. Traditionally, the type of conservation, mitigation or adaptation actions considered suitable for a site/area have been based on a homogenous perception of the vulnerability of a population, species, community or ecosystem (high exposure–high sensitivity–low adaptive capacity). Nonetheless, there are a variety of vulnerability metrics available (Foden et al., 2019), and their relevance for management decisions depends on the paradigm used to assess vulnerability. This dependence means that under the same amount of exposure to a given environmental change, alternative management strategies could be implemented based on the perceived importance of different impact mechanisms. Placing vulnerability metrics in the context of impact mechanisms is one of the innovative aspects of Kling et al. (2020), as it allows us to translate spatial patterns of vulnerability into potential management approaches to climate change for biological scales ranging from a population to an ecosystem. Given the vulnerability conditions considered most relevant at a location, specific actions will be required for the conservation of a species or group of species, or to retain critical biological interactions. Therefore, the conservation, mitigation or adaption strategies to be implemented at a locale depend on the paradigm framing the vulnerability assessment and the impact mechanism considered most critical for the group or biological scale of interest.

Testing and validating how different vulnerability paradigms compare with each other will be an essential next step for the field of global change biology in the coming years. However, measuring vulnerability is still challenging. Some of the challenges are in the form of data limitations. Any vulnerability approach will deliver misrepresentative results if we only consider a small number of environmental drivers and possible responses. So far, the focus on describing annual and seasonal means in temperature and precipitation has meant that vulnerability considerations, regardless of the paradigm, do not consider extremes and variability. The effects of human responses to climate change, or land transformation due to human activities are also not often consider (but see Ordonez, Martinuzzi, Radeloff, & Williams, 2014). There are also limitations with the quantity, quality and availability of biodiversity data that limit the groups and regions for which we can assess biodiversity vulnerability. Even as better environmental and biological data become available, and our confidence in the direction of projected trends of future global change drivers improves further, we will still have significant uncertainties in our vulnerability assessments. Incorporating the uncertainties associated with the data used is a necessary next step in our assessment of biodiversity vulnerability. One way of doing this is to determine the potential of a given exposure level to occur (hazard, cf. Intergovernmental Panel on Climate Change, 2014). Also, by considering the variability in possible impacts, we can generate ‘Maps of ignorance’ for each vulnerability metric. Considering these uncertainties is necessary to translate vulnerability measurements to metrics of risk (probability of harmful consequences, cf. Intergovernmental Panel on Climate Change, 2014) so that adequate management, mitigation and adaption planning can be implemented.

As the threats from climate change, land degradation and invasive species increase in a human‐dominated world, the post‐2020 biodiversity agenda needs to be strategic. Decisions of where and how to invest the limited resources available for conservation need the support of vulnerability assessments grounded on precise impact mechanisms and the best available information. Kling et al. (2020) have taken the first steps in this direction, by highlighting the need to frame vulnerability assessments under a specific paradigm, and the need to analyse the interaction between vulnerability paradigms when defining suitable conservation, management and adaptation strategies.
